# 4-(2-Hydroxy­ethyl)anilinium 3-carb­oxy-4-hydroxy­benzene­sulfonate monohydrate

**DOI:** 10.1107/S1600536809049745

**Published:** 2009-11-25

**Authors:** Graham Smith, Urs D. Wermuth

**Affiliations:** a School of Physical and Chemical Sciences, Queensland University of Technology, GPO Box 2434, Brisbane, Queensland 4001, Australia; b School of Biomolecular and Physical Sciences, Griffith University, Nathan, Queensland 4111, Australia

## Abstract

In the structure of the title compound, C_8_H_12_NO^+^·C_7_H_5_O_6_S^−^·H_2_O, isolated from the reaction of 2-(4-amino­phen­yl)ethanol with 5-sulfosalicylic acid, the cations form head-to-tail hydrogen-bonded chains through *C* ^1^_1_(9) anilinium N^+^—H⋯O_hydrox­yl_ inter­actions while the anions also form parallel but *C* ^1^_1_(8)-linked chains through carboxylic acid O—H⋯O_sulfonate_ inter­actions. These chains inter-associate through a number of N^+^—H⋯O and O—H⋯O bridging inter­actions, giving a two-dimensional array in the *ab* plane.

## Related literature

For the structure of the 2-(4-amino­phen­yl)ethanol salt of 3,5-dinitro­benzoic acid, see: Smith & Wermuth (2009 ▶). For structures of 5-sulfosalicylic acid salts of aniline and substituted anilines, see: Bakasova *et al.* (1991 ▶); Smith (2005 ▶); Smith *et al.* (2005*a* ▶,*b* ▶, 2006 ▶). For hydrogen-bonding graph-set notation, see: Etter *et al.* (1990 ▶).
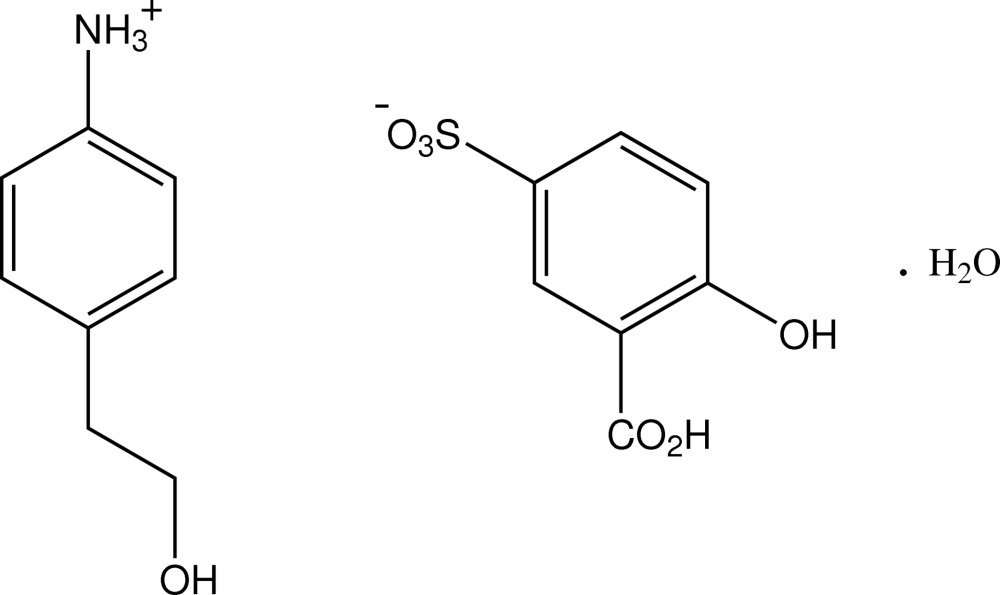


## Experimental

### 

#### Crystal data

C_8_H_12_NO^+^·C_7_H_5_O_6_S^−^·H_2_O
*M* *_r_* = 373.37
Triclinic, 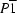

*a* = 7.7412 (6) Å
*b* = 8.7977 (6) Å
*c* = 12.8330 (8) Å
α = 102.169 (6)°
β = 98.538 (6)°
γ = 101.366 (6)°
*V* = 820.97 (11) Å^3^
*Z* = 2
Mo *K*α radiation
μ = 0.24 mm^−1^
*T* = 200 K
0.40 × 0.40 × 0.20 mm

#### Data collection

Oxford Diffraction Gemini-S CCD-detector diffractometer
Absorption correction: multi-scan (**SADABS**; Sheldrick, 1996 ▶) *T*_min_ = 0.934, *T*_max_ = 0.980
10214 measured reflections
3215 independent reflections
2756 reflections with *I* > 2σ(*I*)
*R*_int_ = 0.022

#### Refinement

*R*[*F* ^2^ > 2σ(*F* ^2^)] = 0.032
*wR*(*F* ^2^) = 0.087
*S* = 0.99
3215 reflections
258 parameters
H atoms treated by a mixture of independent and constrained refinement
Δρ_max_ = 0.30 e Å^−3^
Δρ_min_ = −0.30 e Å^−3^

### 

Data collection: *CrysAlis Pro* (Oxford Diffraction (2009 ▶); cell refinement: *CrysAlis Pro*; data reduction: *CrysAlis Pro*; program(s) used to solve structure: *SIR92* (Altomare *et al.*, 1994 ▶); program(s) used to refine structure: *SHELXL97* (Sheldrick, 2008 ▶) within *WinGX* (Farrugia, 1999 ▶); molecular graphics: *PLATON* (Spek, 2009 ▶); software used to prepare material for publication: *PLATON*.

## Supplementary Material

Crystal structure: contains datablocks global, I. DOI: 10.1107/S1600536809049745/tk2580sup1.cif

Structure factors: contains datablocks I. DOI: 10.1107/S1600536809049745/tk2580Isup2.hkl

Additional supplementary materials:  crystallographic information; 3D view; checkCIF report

## Figures and Tables

**Table 1:** Hydrogen-bond geometry (Å, °)

*D*—H⋯*A*	*D*—H	H⋯*A*	*D*⋯*A*	*D*—H⋯*A*
O2—H2⋯O12	0.80 (3)	1.93 (3)	2.6302 (18)	145 (2)
O11—H11⋯O53^i^	0.84 (3)	1.97 (3)	2.8034 (17)	172 (2)
O11*A*—H11*A*⋯O51^ii^	0.81 (2)	1.95 (2)	2.7444 (17)	169 (2)
N4*A*—H41*A*⋯O1*W*	0.94 (2)	1.86 (2)	2.784 (2)	166.5 (19)
N4*A*—H42*A*⋯O11*A* ^iii^	0.89 (2)	1.87 (2)	2.7287 (19)	161 (2)
N4*A*—H43*A*⋯O53	0.94 (2)	1.94 (2)	2.8689 (19)	175.6 (18)
O1*W*—H11*W*⋯O12^iii^	0.87 (3)	2.10 (3)	2.9396 (19)	164 (2)
O1*W*—H12*W*⋯O52^iv^	0.85 (2)	1.92 (2)	2.759 (2)	171 (2)

Symmetry codes: (i) 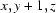; (ii) 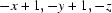; (iii) 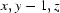; (iv) 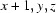.

**Fig. 1.**
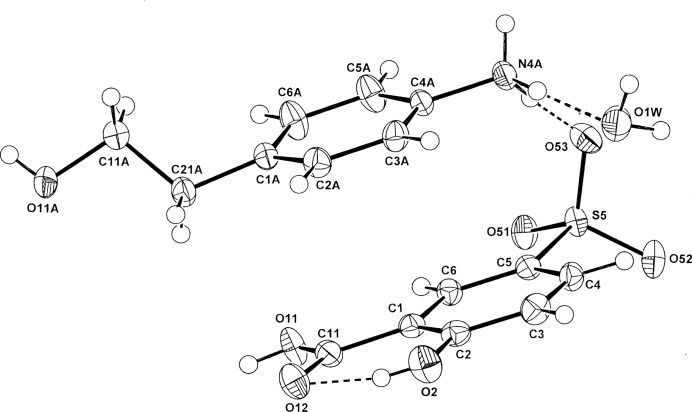
Molecular configuration and atom naming scheme for the substituted anilinium cation, the 5-SSA anion and the water molecule of solvation in (I). Inter-species hydrogen bonds are shown as dashed lines. Displacement ellipsoids are drawn at the 50% probability level.

**Fig. 2.**
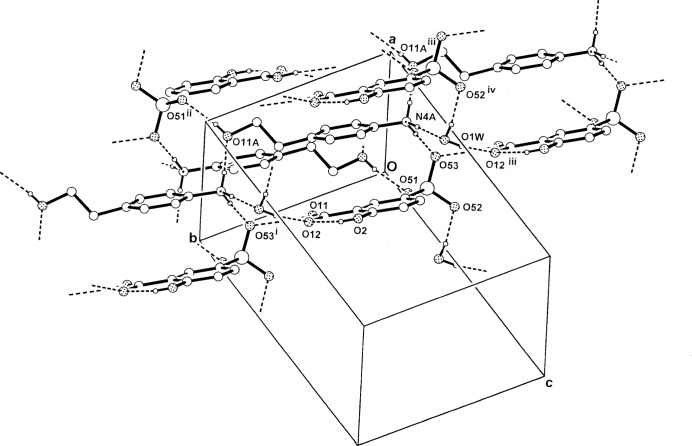
The 2-D hydrogen-bonded array in (I) formed through interlinked hydrogen-bonded cation and anion chains extending in the ab plane. Hydrogen-bonding associations are shown as dashed lines. Non-interacting H atoms are omitted for clarity. For symmetry codes, see Table 1.

## supplementary crystallographic information

### Comment

We recently described the hydrogen bonding in the 1:1 proton-transfer salt of 3,5-dinitrobenzoic acid with 2-(4-aminophenyl)ethanol (Smith & Wermuth, 2009), which was the first reported structure of any compound of this aromatic Lewis base. Because of the common use of 3-carboxy-4-hydroxybenzenesulfonic acid (5-sulfosalicylic acid, 5-SSA) in the formation of stable crystalline compounds of Lewis bases, in particular the analogous aniline (Bakasova *et al.*, 1991), 3-substituted anilines 3-methoxyaniline (Smith *et al.*, 2006), 3-carboxyaniline (Smith, 2005), and the 4-*X*-substituted anilines: *X* = F, Cl, Br (Smith *et al.*, 2005*a*) and *X* = CO_2_H (Smith *et al.*, 2005*b*). We therefore carried out the 1:1 stoichiometric reaction of 5-SSA with this aniline-substituted alcohol in 50% ethanol-water. The result was a 1:1 salt 4-(2-hydroxyethyl)anilinium 3-carboxy-4-hydroxybenzenesulfonate monohydrate, C_8_H_12_NO^+^ C_7_H_5_O_6_S^-^. H_2_O, (I), the structure of which is reported here.

With (I) (Fig. 1), proton transfer occurs and the resulting anilinium group forms head-to-tail hydrogen-bonded cation chains through anilinium N^+^–H···O_hydroxyl_ interactions [graph set C9 (Etter *et al.*, 1990)]. The anions also form similar head-to-tail hydrogen-bonded chains through carboxylic acid O–H···O_sulfonate_ interactions (graph set C8) and lie parallel to the cation chains, extending along the *b* direction. These chains associate through N^+^–H···O_sulfonate_, ···O_carboxyl_, ···O_hydroxyl_ and ···O_water_ interactions as well as through hydroxyl O–H···O_sulfonate_, water O–H···O_sulfonate_ and O–H···O_carboxyl_ bridging interactions (Table 1). The result is a 2-D array (Fig. 2) in which there are also very weak cation–anion aromatic ring π–π interactions [ring centroid separation, 3.8552 (10) Å].

In the 5-SSA anion, the carboxylic acid group is essentially co-planar with the benzene ring [torsion angle C6–C1–C11–O12, -178.40 (15)°] because of the presence of the common intramolecular phenol O–H···O_carboxyl_ hydrogen bond [2.6302 (18) Å].

### Experimental

Compound (I) was synthesized by heating together 1 mmol quantities of 2-(4-aminophenyl)ethanol with 3-carboxy-4-hydroxybenzenesulfonic acid in 50 ml of 50% ethanol–water under reflux for 10 minutes. After concentration to *ca*. 30 ml, partial room temperature evaporation of the hot-filtered solution gave pale-brown plates (m. p. 498 K).

### Refinement

Hydrogen atoms involved in hydrogen-bonding interactions were located by difference methods and their positional and isotropic displacement parameters were refined (see Table 1 for distances). The H-atoms were included in the refinement in calculated positions [C–H(aliphatic) = 0.97 Å and C–H(aromatic) = 0.93 Å) using a riding model approximation, with *U*_iso_(H) = 1.2*U*_eq_(C).

### Crystal data

**Table:**

C_8_H_12_NO^+^·C_7_H_5_O_6_S^−^·H_2_O	*Z* = 2
*M**_r_* = 373.37	*F*(000) = 392
Triclinic, *P*1	*D*_x_ = 1.510 Mg m^−^^3^
Hall symbol: -P 1	Melting point: 498 K
*a* = 7.7412 (6) Å	Mo *K*α radiation, λ = 0.71073 Å
*b* = 8.7977 (6) Å	Cell parameters from 4875 reflections
*c* = 12.8330 (8) Å	θ = 3.2–28.8°
α = 102.169 (6)°	µ = 0.24 mm^−^^1^
β = 98.538 (6)°	*T* = 200 K
γ = 101.366 (6)°	Plate, pale brown
*V* = 820.97 (11) Å^3^	0.40 × 0.40 × 0.20 mm

### Data collection

**Table:**

Oxford Diffraction Gemini-S CCD-detector diffractometer	3215 independent reflections
Radiation source: Enhance (Mo) X-ray source	2756 reflections with *I* > 2σ(*I*)
graphite	*R*_int_ = 0.022
ω scans	θ_max_ = 26.0°, θ_min_ = 3.2°
Absorption correction: multi-scan (*SADABS*; Sheldrick, 1996)	*h* = −9→9
*T*_min_ = 0.934, *T*_max_ = 0.980	*k* = −10→10
10214 measured reflections	*l* = −15→15

### Refinement

**Table:**

Refinement on *F*^2^	Primary atom site location: structure-invariant direct methods
Least-squares matrix: full	Secondary atom site location: difference Fourier map
*R*[*F*^2^ > 2σ(*F*^2^)] = 0.032	Hydrogen site location: inferred from neighbouring sites
*wR*(*F*^2^) = 0.087	H atoms treated by a mixture of independent and constrained refinement
*S* = 0.99	*w* = 1/[σ^2^(*F*_o_^2^) + (0.0575*P*)^2^ + 0.1198*P*] where *P* = (*F*_o_^2^ + 2*F*_c_^2^)/3
3215 reflections	(Δ/σ)_max_ = 0.001
258 parameters	Δρ_max_ = 0.30 e Å^−^^3^
0 restraints	Δρ_min_ = −0.30 e Å^−^^3^

### Special details

**Table:**

Geometry. Bond distances, angles *etc*. have been calculated using the rounded fractional coordinates. All su's are estimated from the variances of the (full) variance-covariance matrix. The cell e.s.d.'s are taken into account in the estimation of distances, angles and torsion angles
Refinement. Refinement of *F*^2^ against ALL reflections. The weighted *R*-factor *wR* and goodness of fit *S* are based on *F*^2^, conventional *R*-factors *R* are based on *F*, with *F* set to zero for negative *F*^2^. The threshold expression of *F*^2^ > σ(*F*^2^) is used only for calculating *R*-factors(gt) *etc*. and is not relevant to the choice of reflections for refinement. *R*-factors based on *F*^2^ are statistically about twice as large as those based on *F*, and *R*- factors based on ALL data will be even larger.

### Fractional atomic coordinates and isotropic or equivalent isotropic displacement parameters (Å^2^)

**Table:**

	*x*	*y*	*z*	*U*_iso_*/*U*_eq_	
O11A	0.80608 (18)	0.87360 (14)	0.00089 (10)	0.0341 (4)	
N4A	0.78541 (19)	0.07130 (16)	0.19112 (12)	0.0234 (4)	
C1A	0.8163 (2)	0.53103 (17)	0.13072 (12)	0.0212 (4)	
C2A	0.9359 (2)	0.51052 (18)	0.21625 (12)	0.0244 (4)	
C3A	0.9278 (2)	0.35998 (18)	0.23691 (13)	0.0241 (5)	
C4A	0.7995 (2)	0.23068 (17)	0.17042 (12)	0.0206 (4)	
C5A	0.6827 (2)	0.24656 (19)	0.08278 (14)	0.0299 (5)	
C6A	0.6921 (2)	0.39667 (19)	0.06356 (14)	0.0304 (5)	
C11A	0.8410 (2)	0.72115 (19)	0.00469 (14)	0.0302 (5)	
C21A	0.8142 (2)	0.69635 (18)	0.11528 (13)	0.0247 (4)	
S5	0.31926 (5)	0.00829 (4)	0.26512 (3)	0.0203 (1)	
O2	0.83804 (16)	0.60833 (15)	0.51275 (10)	0.0334 (4)	
O11	0.43638 (16)	0.62380 (14)	0.25827 (10)	0.0310 (4)	
O12	0.67893 (15)	0.76007 (13)	0.38590 (10)	0.0323 (4)	
O51	0.17800 (14)	0.04515 (12)	0.19312 (9)	0.0253 (3)	
O52	0.25277 (15)	−0.07135 (13)	0.34506 (9)	0.0289 (4)	
O53	0.43041 (14)	−0.08330 (13)	0.20599 (9)	0.0292 (3)	
C1	0.5791 (2)	0.47935 (17)	0.36674 (12)	0.0205 (4)	
C2	0.7134 (2)	0.47689 (18)	0.45347 (12)	0.0226 (4)	
C3	0.7206 (2)	0.33328 (19)	0.48234 (13)	0.0257 (5)	
C4	0.5988 (2)	0.19310 (18)	0.42610 (12)	0.0227 (5)	
C5	0.46674 (19)	0.19342 (17)	0.33820 (12)	0.0192 (4)	
C6	0.45641 (19)	0.33538 (17)	0.30967 (12)	0.0201 (4)	
C11	0.5704 (2)	0.63262 (18)	0.33854 (13)	0.0233 (5)	
O1W	0.9577 (2)	0.04283 (16)	0.39011 (11)	0.0352 (4)	
H2A	1.02240	0.59840	0.26020	0.0290*	
H3A	1.00740	0.34710	0.29440	0.0290*	
H5A	0.59910	0.15770	0.03750	0.0360*	
H6A	0.61400	0.40810	0.00480	0.0370*	
H11A	0.824 (3)	0.894 (3)	−0.0554 (19)	0.047 (6)*	
H12A	0.75870	0.63730	−0.05290	0.0360*	
H13A	0.96310	0.71970	−0.00400	0.0360*	
H21A	0.90780	0.77360	0.17060	0.0300*	
H22A	0.70000	0.71900	0.12720	0.0300*	
H41A	0.858 (3)	0.075 (2)	0.2578 (17)	0.040 (5)*	
H42A	0.818 (3)	0.009 (3)	0.1367 (19)	0.049 (6)*	
H43A	0.668 (3)	0.026 (2)	0.1963 (15)	0.035 (5)*	
H2	0.823 (3)	0.685 (3)	0.491 (2)	0.062 (8)*	
H3	0.80840	0.33250	0.54000	0.0310*	
H4	0.60390	0.09830	0.44620	0.0270*	
H6	0.36750	0.33520	0.25230	0.0240*	
H11	0.434 (3)	0.715 (3)	0.2491 (17)	0.047 (6)*	
H11W	0.883 (4)	−0.035 (3)	0.403 (2)	0.069 (8)*	
H12W	1.047 (3)	0.002 (3)	0.3819 (17)	0.046 (6)*	

### Atomic displacement parameters (Å^2^)

**Table:**

	*U*^11^	*U*^22^	*U*^33^	*U*^12^	*U*^13^	*U*^23^
O11A	0.0568 (8)	0.0265 (6)	0.0266 (7)	0.0153 (6)	0.0133 (6)	0.0147 (5)
N4A	0.0250 (7)	0.0188 (7)	0.0293 (8)	0.0067 (6)	0.0076 (6)	0.0093 (6)
C1A	0.0239 (8)	0.0180 (7)	0.0226 (8)	0.0051 (6)	0.0050 (6)	0.0066 (6)
C2A	0.0268 (8)	0.0188 (7)	0.0228 (8)	−0.0002 (6)	0.0006 (6)	0.0028 (6)
C3A	0.0250 (8)	0.0252 (8)	0.0217 (8)	0.0058 (6)	0.0000 (6)	0.0085 (6)
C4A	0.0227 (8)	0.0164 (7)	0.0257 (8)	0.0067 (6)	0.0079 (6)	0.0076 (6)
C5A	0.0284 (9)	0.0190 (8)	0.0356 (9)	0.0014 (7)	−0.0066 (7)	0.0054 (7)
C6A	0.0290 (9)	0.0230 (8)	0.0341 (9)	0.0041 (7)	−0.0094 (7)	0.0087 (7)
C11A	0.0428 (10)	0.0227 (8)	0.0291 (9)	0.0100 (7)	0.0133 (8)	0.0088 (7)
C21A	0.0302 (8)	0.0182 (7)	0.0250 (8)	0.0045 (6)	0.0040 (7)	0.0062 (6)
S5	0.0188 (2)	0.0172 (2)	0.0251 (2)	0.0033 (1)	0.0016 (2)	0.0087 (2)
O2	0.0322 (7)	0.0256 (6)	0.0333 (7)	−0.0010 (5)	−0.0059 (5)	0.0035 (5)
O11	0.0337 (7)	0.0180 (6)	0.0393 (7)	0.0060 (5)	−0.0028 (5)	0.0102 (5)
O12	0.0320 (6)	0.0204 (6)	0.0399 (7)	0.0000 (5)	0.0026 (5)	0.0062 (5)
O51	0.0224 (6)	0.0253 (6)	0.0276 (6)	0.0033 (5)	−0.0010 (5)	0.0114 (5)
O52	0.0258 (6)	0.0283 (6)	0.0352 (7)	0.0027 (5)	0.0032 (5)	0.0191 (5)
O53	0.0270 (6)	0.0211 (5)	0.0377 (7)	0.0061 (5)	0.0065 (5)	0.0032 (5)
C1	0.0206 (7)	0.0209 (7)	0.0212 (7)	0.0058 (6)	0.0071 (6)	0.0049 (6)
C2	0.0216 (7)	0.0229 (8)	0.0211 (8)	0.0031 (6)	0.0050 (6)	0.0023 (6)
C3	0.0232 (8)	0.0311 (8)	0.0225 (8)	0.0070 (7)	0.0000 (6)	0.0085 (7)
C4	0.0248 (8)	0.0234 (8)	0.0236 (8)	0.0085 (6)	0.0053 (6)	0.0106 (6)
C5	0.0179 (7)	0.0193 (7)	0.0211 (7)	0.0044 (6)	0.0053 (6)	0.0055 (6)
C6	0.0196 (7)	0.0205 (7)	0.0211 (7)	0.0064 (6)	0.0027 (6)	0.0066 (6)
C11	0.0236 (8)	0.0199 (8)	0.0271 (8)	0.0056 (6)	0.0075 (7)	0.0054 (6)
O1W	0.0372 (8)	0.0354 (7)	0.0397 (7)	0.0111 (6)	0.0129 (6)	0.0178 (6)

### Geometric parameters (Å, °)

**Table:**

S5—O51	1.4563 (12)	C4A—C5A	1.384 (2)
S5—O52	1.4581 (12)	C5A—C6A	1.384 (2)
S5—O53	1.4747 (12)	C11A—C21A	1.518 (2)
S5—C5	1.7723 (16)	C2A—H2A	0.9300
O11A—C11A	1.429 (2)	C3A—H3A	0.9300
O11A—H11A	0.81 (2)	C5A—H5A	0.9300
O2—C2	1.348 (2)	C6A—H6A	0.9300
O11—C11	1.327 (2)	C11A—H12A	0.9700
O12—C11	1.234 (2)	C11A—H13A	0.9700
O2—H2	0.80 (3)	C21A—H21A	0.9700
O11—H11	0.84 (3)	C21A—H22A	0.9700
O1W—H11W	0.87 (3)	C1—C2	1.413 (2)
O1W—H12W	0.85 (2)	C1—C11	1.479 (2)
N4A—C4A	1.468 (2)	C1—C6	1.404 (2)
N4A—H41A	0.94 (2)	C2—C3	1.398 (2)
N4A—H42A	0.89 (2)	C3—C4	1.377 (2)
N4A—H43A	0.94 (2)	C4—C5	1.406 (2)
C1A—C6A	1.393 (2)	C5—C6	1.387 (2)
C1A—C2A	1.394 (2)	C3—H3	0.9300
C1A—C21A	1.512 (2)	C4—H4	0.9300
C2A—C3A	1.396 (2)	C6—H6	0.9300
C3A—C4A	1.379 (2)		
			
O51—S5—O53	113.02 (7)	C5A—C6A—H6A	119.00
O51—S5—C5	106.82 (7)	C1A—C6A—H6A	119.00
O52—S5—O53	110.32 (7)	C21A—C11A—H12A	110.00
O52—S5—C5	107.10 (7)	C21A—C11A—H13A	110.00
O53—S5—C5	105.72 (7)	H12A—C11A—H13A	109.00
O51—S5—O52	113.33 (7)	O11A—C11A—H12A	110.00
C11A—O11A—H11A	109.8 (18)	O11A—C11A—H13A	110.00
C2—O2—H2	109.8 (18)	C1A—C21A—H22A	108.00
C11—O11—H11	110.7 (16)	C1A—C21A—H21A	108.00
H11W—O1W—H12W	102 (3)	H21A—C21A—H22A	107.00
H41A—N4A—H43A	105.5 (18)	C11A—C21A—H21A	108.00
H41A—N4A—H42A	110 (2)	C11A—C21A—H22A	108.00
C4A—N4A—H41A	112.1 (11)	C2—C1—C11	119.52 (14)
C4A—N4A—H43A	111.1 (12)	C6—C1—C11	121.67 (14)
H42A—N4A—H43A	110 (2)	C2—C1—C6	118.82 (14)
C4A—N4A—H42A	108.5 (17)	O2—C2—C3	116.62 (14)
C2A—C1A—C6A	118.47 (14)	C1—C2—C3	120.02 (14)
C2A—C1A—C21A	120.69 (14)	O2—C2—C1	123.35 (14)
C6A—C1A—C21A	120.74 (14)	C2—C3—C4	120.56 (15)
C1A—C2A—C3A	120.99 (14)	C3—C4—C5	119.94 (15)
C2A—C3A—C4A	118.74 (14)	C4—C5—C6	120.10 (14)
N4A—C4A—C5A	118.38 (14)	S5—C5—C4	118.07 (12)
C3A—C4A—C5A	121.53 (15)	S5—C5—C6	121.82 (12)
N4A—C4A—C3A	120.08 (14)	C1—C6—C5	120.53 (14)
C4A—C5A—C6A	119.01 (15)	O11—C11—O12	121.98 (15)
C1A—C6A—C5A	121.20 (15)	O11—C11—C1	115.13 (14)
O11A—C11A—C21A	106.35 (13)	O12—C11—C1	122.89 (14)
C1A—C21A—C11A	115.37 (13)	C4—C3—H3	120.00
C1A—C2A—H2A	120.00	C2—C3—H3	120.00
C3A—C2A—H2A	119.00	C3—C4—H4	120.00
C4A—C3A—H3A	121.00	C5—C4—H4	120.00
C2A—C3A—H3A	121.00	C1—C6—H6	120.00
C4A—C5A—H5A	121.00	C5—C6—H6	120.00
C6A—C5A—H5A	120.00		
			
O52—S5—C5—C4	48.08 (14)	O11A—C11A—C21A—C1A	170.97 (13)
O52—S5—C5—C6	−133.01 (13)	C6—C1—C2—O2	179.56 (14)
O53—S5—C5—C4	−69.56 (13)	C6—C1—C2—C3	−1.1 (2)
O53—S5—C5—C6	109.35 (13)	C11—C1—C2—O2	−0.7 (2)
O51—S5—C5—C6	−11.28 (15)	C11—C1—C2—C3	178.66 (14)
O51—S5—C5—C4	169.81 (12)	C2—C1—C6—C5	0.2 (2)
C6A—C1A—C21A—C11A	−59.7 (2)	C11—C1—C6—C5	−179.57 (14)
C2A—C1A—C21A—C11A	123.94 (16)	C2—C1—C11—O11	−177.96 (14)
C6A—C1A—C2A—C3A	−2.3 (2)	C2—C1—C11—O12	1.9 (2)
C21A—C1A—C2A—C3A	174.16 (15)	C6—C1—C11—O11	1.8 (2)
C2A—C1A—C6A—C5A	2.1 (2)	C6—C1—C11—O12	−178.40 (15)
C21A—C1A—C6A—C5A	−174.34 (15)	O2—C2—C3—C4	−179.87 (14)
C1A—C2A—C3A—C4A	0.5 (2)	C1—C2—C3—C4	0.7 (2)
C2A—C3A—C4A—C5A	1.6 (2)	C2—C3—C4—C5	0.6 (2)
C2A—C3A—C4A—N4A	−178.94 (14)	C3—C4—C5—S5	177.44 (12)
N4A—C4A—C5A—C6A	178.74 (15)	C3—C4—C5—C6	−1.5 (2)
C3A—C4A—C5A—C6A	−1.8 (2)	S5—C5—C6—C1	−177.77 (12)
C4A—C5A—C6A—C1A	−0.1 (2)	C4—C5—C6—C1	1.1 (2)

### Hydrogen-bond geometry (Å, °)

**Table:**

*D*—H···*A*	*D*—H	H···*A*	*D*···*A*	*D*—H···*A*
O2—H2···O12	0.80 (3)	1.93 (3)	2.6302 (18)	145 (2)
O11—H11···O53^i^	0.84 (3)	1.97 (3)	2.8034 (17)	172 (2)
O11A—H11A···O51^ii^	0.81 (2)	1.95 (2)	2.7444 (17)	169 (2)
N4A—H41A···O1W	0.94 (2)	1.86 (2)	2.784 (2)	166.5 (19)
N4A—H42A···O11A^iii^	0.89 (2)	1.87 (2)	2.7287 (19)	161 (2)
N4A—H43A···O53	0.94 (2)	1.94 (2)	2.8689 (19)	175.6 (18)
O1W—H11W···O12^iii^	0.87 (3)	2.10 (3)	2.9396 (19)	164 (2)
O1W—H12W···O52^iv^	0.85 (2)	1.92 (2)	2.759 (2)	171 (2)
C3A—H3A···O2^v^	0.93	2.50	3.373 (2)	157
C6—H6···O51	0.93	2.57	2.9437 (19)	104

Symmetry codes: (i) *x*, *y*+1, *z*; (ii) −*x*+1, −*y*+1, −*z*; (iii) *x*, *y*−1, *z*; (iv) *x*+1, *y*, *z*; (v) −*x*+2, −*y*+1, −*z*+1.
